# Cholic Acid-Conjugated Methylcellulose-Polyethylenimine Nano-Aggregates for Drug Delivery Systems

**DOI:** 10.3390/nano9030459

**Published:** 2019-03-19

**Authors:** Taewan Kim, Jaehong Park, Tae-il Kim

**Affiliations:** 1 Department of Biosystems & Biomaterials Science and Engineering, College of Agriculture and Life Sciences, Seoul National University, 1 Gwanak-ro, Gwanak-gu, Seoul 08826, Korea; tewhan32321@naver.com (T.K.); pjh0520@snu.ac.kr (J.P.); 2 Research Institute of Agriculture and Life Sciences, Seoul National University, 1 Gwanak-ro, Gwanak-gu, Seoul 08826, Korea

**Keywords:** cholic acid, methylcellulose, polyethylenimine, molecular conformation, nano-aggregates, drug delivery systems

## Abstract

Cholic acid-conjugated methylcellulose-polyethylenimines (MCPEI-CAs) were synthesized and characterized for drug delivery systems. Their synthesis was confirmed by ^1^H NMR and FT-IR analysis. Induced circular dichroism result with Congo red showed that methylcellulose (MC) and polyethylenimine-grafted cationic derivative (MC-PEI) would have helical conformation and random coil structure, respectively. It was found that MCPEI-CAs could form positively charged (>30 mV Zeta-potential) and spherical nano-aggregates (~250 nm Z-average size) by hydrophobic interaction of CA moieties. Critical aggregation concentration of MCPEI-CA_10_ was measured as 7.2 × 10^−3^ mg/mL. MCPEI-CA_10_ could encapsulate the anticancer drug doxorubicin (Dox) with 58.0% of drug loading content and 23.2% of drug loading efficiency and its release was facilitated in acidic condition. Cytotoxicity of MCPEI-CAs was increased with the increase of cholic acid (CA) graft degrees, probably due to the cellular membrane disruption by interaction with specific molecular structure of amphiphilic MCPEI-CA nano-aggregates. MCPEI-CA_10_/Dox nano-aggregates showed concentration-dependent anticancer activity, which could overcome the multidrug resistance of cancer cells. In this work, molecular conformation change of MC derivatives by chemical modification and a potential of MCPEI-CA_10_/Dox nano-aggregates for drug delivery systems were revealed.

## 1. Introduction

Nanoparticle-based drug delivery systems have been extensively studied during several decades [1,2,3]. They have lots of advantages, such as applicability for various administration routes (oral, parenteral administration, etc.), facile drug delivery in body due to their nano-sizes, stabilization of loaded drug molecules, and sustained or controlled drug release.

Therefore, various types of nanoparticles have been developed for drug delivery systems, including dendrimers [4], liposomes [5], polymeric micelles [6], silica nanoparticles [7], carbon nanomaterials [8], gold nanoparticles [9], and magnetic nanoparticles [10]. Among them, biopolymers and biomolecules were utilized in order to introduce their innate bioactivity and improve the biocompatibility of the delivery systems [11,12]. Polysaccharides are one of the candidates for efficient drug delivery systems because of their high biocompatibility, biodegradability, chemically modifiable various functional groups, and abundant and replenishable sources [13,14].

Methylcellulose (MC) is a cellulose ether derivative containing several methylated hydroxyl groups (degree of methylation: 1.4–2.5) [15,16]. MC can form physical gels in aqueous condition by intra- and intermolecular hydrophobic interactions, which is dependent on the degree of methylation, molecular weight, and temperature. Usually, MC has been used for emulsifying agents, food thickeners, and hydrogels for drug delivery systems due to the high biocompatibility and thermo-responsive sol-gel transition behavior. Recently, polyethylenimine-grafted cationic MC derivative (MC-PEI) has been reported as an efficient gene delivery system with serum-compatibility and endosome buffering ability [17].

Cholic acid (CA) is a primary bile acid, which is produced from cholesterol in liver [18]. Interestingly, it shows an amphiphilicity (critical micelle concentration: 18.4 mM) due to its unique chemical structure, hydrophobic steroid body, and spatially segregated hydrophilic hydroxyl groups, acting as solubilizers or emulsifiers for dissolution of lipids and cholesterols in body [19]. Therefore, CA-modified drug/gene delivery systems have been developed, encapsulating hydrophobic drug molecules by CA amphiphilicity or enhancing their intestinal absorption rate via CA transporter-mediated uptake [20,21,22,23].

In this work, we synthesized cholic acid-conjugated methylcellulose-polyethylenimines (MCPEI-CAs) with various CA graft degrees in order to take advantages of MC and CA and characterized for the anticancer drug doxorubicin (Dox) delivery systems. In particular, molecular conformation change of MC and MC-PEI by chemical modification and nano-aggregates formation behavior by MCPEI-CA molecules were analyzed. Interaction of MCPEI-CA nano-aggregates with cellular membrane was also examined, and finally, a potential of MCPEI-CA nano-aggregates was revealed for drug delivery systems.

## 2. Materials and Methods

### 2.1. Materials

Methylcellulose (MC, 15 cP, 2% in H_2_O), polyethylenimine (PEI, molecular weight 0.8 k and 25 kDa), 3-[4,5-dimethylthiazol-2-yl]-2,5-diphenyltetrazolium bromide (MTT), pyrene, *N*,*N*′-dicyclohexylcarbodiimide (DCC), *N*-hydroxysuccinimide (NHS), and Congo red (CR) were purchased from Sigma-Aldrich (St. Louis, MO, USA). Doxorubicin hydrochloride (Dox HCl) was purchased from Wako Pure Chemical Industries (Osaka, Japan). Cholic acid (CA) was purchased from Tokyo Chemical Industry (Tokyo, Japan). Sodium periodate and sodium tetrahydroborate were purchased from Junsei Chemical (Tokyo, Japan). Dulbecco’s modified Eagle’s medium (DMEM), Dulbecco’s phosphate buffered saline (DPBS), 0.25% Trypsin-ethylenediaminetetraacetic acid (EDTA) and fetal bovine serum (FBS) were purchased from Invitrogen (Carlsbad, CA, USA). All other chemicals were purchased and used without any further purification.

### 2.2. Synthesis and Characterization of Cholic Acid-conjugated Methylcellulose-polyethylenimines (MCPEI-CAs)

First, MC-PEI was synthesized as a template polymer according to the previous work in Reference [17]. Briefly, MC was oxidized to oxidized methylcellulose (OXMC) using sodium periodate and PEI0.8k was grafted onto OXMC, followed by reduction by sodium tetrahydroborate through reductive amination. After dialysis and lyophilization, MC-PEI was obtained.

In order to conjugate CA to MC-PEI, carboxylic acid group of CA was activated to NHS ester by using the DCC coupling method. CA and NHS were dissolved in tetrahydrofuran (THF) and DCC/THF solution was added to the CA/NHS mixture solution. After 4 h of reaction at room temperature in dark condition and the followed 2 times of washing step with cold n-hexane, CA-NHS ester was obtained.

For the synthesis of MCPEI-CA, previously synthesized MC-PEI and CA-NHS ester were dissolved in DMSO and reacted with each other for 1 day at room temperature under nitrogen atmosphere (CA-NHS/primary amine of PEI0.8k = 3.6, 6, or 10 molar feed ratio). Then, 2 times of ethyl ether precipitation and 1 day of dialysis (MWCO = 6–8 kDa) against ultrapure water were performed. After lyophilization, MCPEI-CA_3_, MCPEI-CA_6_, or MCPEI-CA_10_ was obtained, respectively.

Each step of MCPEI-CA synthesis was confirmed by ^1^H NMR (400 MHz JNM-LA400, JEOL, Japan or 600 MHz AVANCE 600, Bruker, Billerica, MA, USA) with D_2_O solvent. Fourier Transform Infrared Spectrometry (FT-IR) spectra of the synthesized polymers were also recorded by ATR FT-IR spectrometer (Nicolet 6700, Thermo Scientific, Waltham, MA, USA). The polymer samples were analyzed in the range of 600–4000 cm^−1^ with 4 cm^−1^ interval.

### 2.3. Measurement of Critical Aggregation Concentration (CAC)

In order to characterize CAC of MCPEI-CA, pyrene absorbance method was used, as previously reported [24]. 20 μL of pyrene ethanol solutions (0.1 mM) were dried in vials under vacuum overnight in dark condition. 1 mL of MCPEI-CA water solutions with various concentrations were added to the formed pyrene film in vials, giving the final pyrene a concentration of 6 × 10^−7^ M. After sonication for 10 min and gentle mixing (dark condition) of MCPEI-CA/pyrene solutions at room temperature for 24 h, absorbance of pyrene was measured by UV-Vis spectrophotometer using quartz cuvettes (200–400 nm). The total absorbances by the sum of four absorbance values (242, 272, 320, and 336 nm) were plotted against MCPEI-CA concentrations. CAC of MCPEI-CA sample was determined by setting to the center of the plot sigmoid.

### 2.4. Encapsulation of Dox in MCPEI-CA Nano-aggregates

Dox HCl solution (5 mg/mL, DMSO) was mixed with 2 equivalent moles of triethylamine for removal of HCl salt. After 1 day, the Dox solution was mixed with MCPEI-CA solution (DMSO) for 1.5 h. Then, the MCPEI-CA/Dox solution (DMSO) was added dropwise to water for 4 h. The mixture solution was dialyzed against ultrapure water with dialysis membrane (MWCO = 2k) for 6 h and lyophilized, leaving MCPEI-CA/Dox nano-aggregates. All procedures were performed in dark condition at 25 °C.

### 2.5. Evaluation of Dox Loading in Cholic Acid-conjugated Methylcellulose-polyethylenimines (MCPEI-CA)/Doxorubicin (Dox) Nano-aggregates

Previously prepared MCPEI-CA/Dox was dissolved in DMSO and the absorbance of Dox was measured by a microplate reader (Synergy H1, BioTek, Winooski, VT, USA) at 485 nm. Loaded Dox amount was determined according to the previously prepared calibration curve of Dox solution (DMSO). Drug loading content (DLC) and drug loading efficiency (DLE) were calculated according to the following formula.

(1) $$\mathrm{DLC}\;(\%)=\frac{Weight\;of\;Loaded\;DOX}{Weight\;of\;MCPEI-CA}\times 100$$

(2) $$\mathrm{DLE}\;(\%)=\frac{Weight\;of\;Loaded\;DOX}{Weight\;of\;total\;DOX\;for\;loading}\times 100$$

### 2.6. Average Particle Size and Zeta-Potential Measurements

Z-average particle sizes of MCPEI-CA and MCPEI-CA/Dox nanoaggregates were measured by a Zeta-sizer Nano ZS90 (Malvern Instruments, Malvern, UK) with He-Ne laser beam (633 nm) at 25 °C. MCPEI-CA and MCPEI-CA/Dox water solutions (10, 20, and 40 μg/mL) were used for measurements. Zeta-potential values were also measured by a Zeta-sizer. All measurements were performed 3 times.

### 2.7. Transmission Electron Microscopy (TEM)

The morphology of MCPEI-CA and MCPEI-CA/Dox nano-aggregates was observed by Energy-filtering transmission electron microscopy (EF-TEM, LIBRA 120, Carl Zeiss, Oberkochen, Germany). MCPEI-CA and MCPEI-CA/Dox water solutions (10 and 20 μg/mL) were prepared and deposited on TEM copper grid plates 4 times. The samples were then stained with filtered uranium acetate solution for 10 s. After careful removal of residual solutions, the images were visualized with an accelerating voltage of 120 kV.

### 2.8. Release Profile of Doxorubicin (Dox) from Cholic Acid-conjugated Methylcellulose-polyethylenimines (MCPEI-CA) Nano-aggregates

MCPEI-CA/Dox was dissolved in sodium acetate buffer (pH 5.4) and HEPES buffer (pH 7.4). The solutions were dialyzed on an orbital shaker against identical buffers with dialysis membrane (MWCO = 8k) at 37 °C in dark condition. 500 μL of buffer solutions were collected at several time points and the same volume of fresh buffer was replenished. The absorbance of Dox in the collected buffer was measured by a microplate reader at 485 nm. Released Dox amount was determined according to the previously prepared calibration curve of Dox solution.

### 2.9. Circular Dichroism (CD) Measurements

CD measurements were performed according to the previous report [25]. Polymer solutions (water, 15.4 × 10^−3^ mol repeat unit/L) and Congo red solution (water, 1.0 × 10^−3^ mol/L) were prepared. Equal volume (50 μL) of each polymer solution and Congo red solution were mixed for 1 h at room temperature to give a dye molecule to repeat unit ratio of 1:15.4. Then, CD spectra were recorded using Circular Dichroism Detector (Chirascan plus, Applied Photophysics, Surrey, UK) with slit width of 1 nm (190–250 nm range).

### 2.10. Congo Red Absorbance Measurements

Polymer solutions (water, 1.54 × 10^−3^ mol repeat unit/L) and Congo red solution (water, 0.1 × 10^−3^ mol/L) were prepared. Equal volume (500 μL) of each polymer solution and Congo red solution were mixed for 1 h at room temperature to give a dye molecule to repeat unit ratio of 1:15.4. Then, Congo red absorbance of the solutions was recorded using UV-Vis spectrophotometer (Optizen POP BIO, K LAB, Daejeon, Korea) using quartz cuvettes (430–600 nm).

### 2.11. Cell Culture

Human cervical adenocarcinoma cells (HeLa) and human lung adenocarcinoma epithelial cells (A549) were maintained in a 5% CO_2_ incubator at 37 °C in DMEM + GlutaMax-I medium, which was supplemented with 10% FBS and 1% penicillin/streptomycin.

### 2.12. MTT Assay

MTT assay was performed to examine the cytotoxicity of MCPEI-CAs. PEI25k was used as a control. Cells were seeded on a 96-well plate at a density of 1.0 × 10^4^ cells/well, respectively. Having achieved 70–80% confluence after 1 day, the cells were exposed to 100 μL polymer solutions (serum-free medium) with various concentrations for 4 h. Subsequently, the media was changed with fresh medium (10% FBS). After 24 h, the cells were treated with 25 μL of MTT stock solution (2 mg/mL in DPBS) and incubated for 2 h at 37 °C. After removing each medium carefully, 150 μL of DMSO was added to each well to dissolve the formazan crystal formed by proliferating cells. The absorbance was measured at 570 nm using a microplate reader. Results were presented as relative cell viabilities (RCV, percentage values relative to value of untreated control cells). All experiments were performed in triplicate.

### 2.13. Lactate Dehydrogenase (LDH) Assay

Cells were seeded on a 96-well culture plate at a density of 0.5 × 10^4^ cells/well. After achieving 70–80% confluence, the cells were treated with MCPEI-CA solutions (DMEM) with a series of concentration for 4 h. Then, the media (100 μL) was collected and LDH assay was performed by LDH Cytotoxicity Detection Kit (Takara Biochemicals, Shiga, Japan). Measured LDH level at 490 nm was normalized in terms of percentage to the value of positive control (1.0% Triton-X100). The assay was performed in triplicate.

### 2.14. Anticancer Activity of Cholic Acid-conjugated Methylcellulose-polyethylenimines (MCPEI-CA)/Doxorubicin (Dox) Nano-aggregates

Anticancer activity of MCPEI-CA/Dox was assessed by MTT assay. Cells were seeded in a 96-well plate at a density of 1.0 × 10^4^ cells/well. Having achieved 70–80% confluence after 1 day, the cells were exposed to MCPEI-CA/Dox solution (DMEM) with various Dox concentrations for 4 h. Free Dox solution was used as a control. Subsequently, the media was changed with fresh medium (10% FBS). After 24 h of incubation, MTT assay was performed in an identical manner with above procedures.

## 3. Results and Discussion

### 3.1. Synthesis and Characterization of Cholic Acid-conjugated Methylcellulose-polyethylenimines (MCPEI-CAs)

First, MC was oxidized by using periodate ion (IO_4_^−^) to form aldehyde groups for conjugation of PEI0.8k according to the previous report [17]. It has been known that periodate ions can cleave the carbon-carbon bond between diol (here, C2 carbon and C3 carbon of methylcellulose anhydroglucose unit) by forming dialdehyde [26]. Then, PEI0.8k was conjugated to oxidized methylcellulose (OXMC) via nucleophilic attack of primary amines of PEI0.8k to aldehyde groups followed by reduction of formed imine to secondary amine, synthesizing MC-PEI. Carboxyl group of CA was activated to NHS ester (CA-NHS) by using DCC/NHS coupling method. Insoluble reaction product, dicyclohexylurea (DCU) was removed by n-hexane precipitation. Then, activated CA was conjugated to MC-PEI by amide bond formation between primary amine of PEI and CA-NHS. The synthesis scheme was shown in Figure S1.

**Figure 1:**
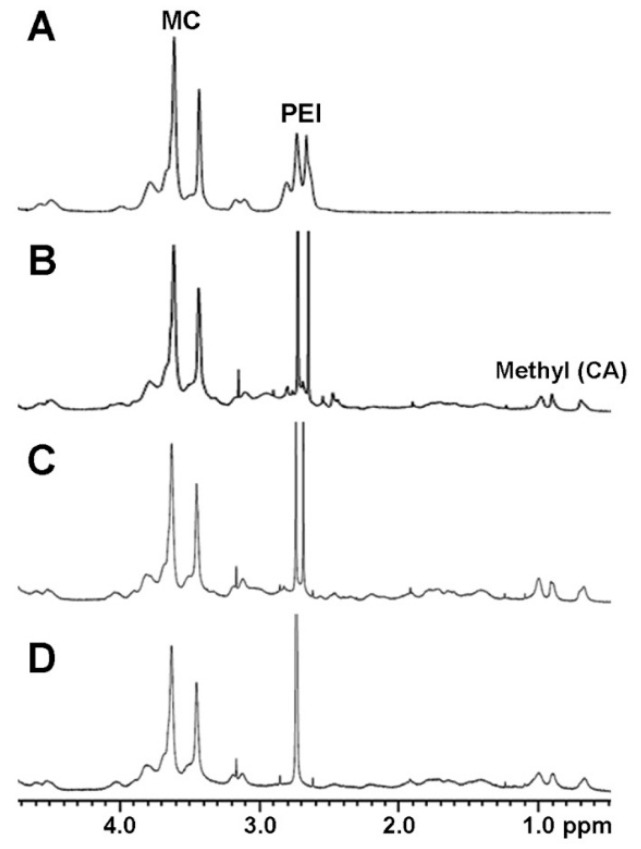
^1^H NMR spectra of the synthesized polymers. (**A**) polyethylenimine-grafted cationic MC derivative (MC-PEI), (**B**) cholic acid-conjugated methylcellulose-polyethylenimines (MCPEI-CA)_3_, (**C**) MCPEI-CA_6_, and (**D**) MCPEI-CA_10_.

The synthesis of MCPEI-CAs was confirmed by ^1^H NMR. In Figure 1A, proton peaks from MC (3.0–4.6 ppm) and from PEI0.8k (2.6–2.8 ppm) were observed in the NMR result of MC-PEI. By comparing the integral of MC protons and PEI protons, it was calculated that PEI0.8k was conjugated to every 14.2 anhydroglucose unit of MC and that the degree of PEI graft was 7.04%.

After reaction with CA-NHS, new proton peaks from three methyl groups of CA (0.7–1.0 ppm) appeared, which means that CA was successfully conjugated to MC-PEI (Figure 1B–D). The integral of CA protons was increased with the increase of initial feed ratio. The degree of CA graft was calculated by comparing the integral of CA methyl protons and other residual protons (Table 1). MCPEI-CAs were named after initial feed ratio between PEI and CA. About 50% of added CA was found to be conjugated to MC-PEI.

Conjugation of CA to MC-PEI was also confirmed by FT-IR analysis (Figure S2). In MC-PEI result, C–H stretching vibration peak (2870–3000 cm^−1^), N-H bending peak (PEI, 1720 cm^−1^), and N–H wagging peak (PEI, 810 cm^−1^) were observed. After conjugation of CA, new peaks from C=O stretching peak (amide, 1600–1690 cm^−1^) and from N-H bending peak (amide, 1500–1580 cm^−1^) were found, meaning formation of amide bond between PEI and CA. In addition, methyl stretching peak (CA, 2580 cm^−1^) and methyl rocking peaks (CA, 975 cm^−1^ and 1075 cm^−1^) also appeared. These results showed that MCPEI-CAs were successfully synthesized.

### 3.2. Characterization of Cholic Acid-conjugated Methylcellulose-Polyethylenimines (MCPEI-CA) Nano-aggregates for Drug Delivery Systems

In order to examine the hydrophobic drug encapsulating ability of MCPEI-CA, critical aggregation concentration (CAC) was measured by pyrene absorbance method [24]. As a result, CACs of MCPEI-CA_3_, MCPEI-CA_6_, and MCPEI-CA_10_ were determined as 2.0 × 10^−2^ mg/mL, 1.5 × 10^−2^ mg/mL, and 7.2 × 10^−3^ mg/mL, respectively. The more grafted cholic acid was, the lower CAC MCPEI-CA showed, due to the improved nano-aggregates formation by hydrophobicity of CA. Based on this result, only MCPEI-CA_10_ was further characterized for drug delivery systems, which has the lowest CAC among the derivatives.

DLE and DLC of MCPEI-CA_10_ were measured, using hydrophobic anticancer agent, Dox. Its DLE and DLC were determined as 58.0% and 23.2% respectively via dialysis method, showing that MCPEI-CA_10_ could encapsulate Dox molecules efficiently for drug delivery systems. It was postulated that MCPEI-CA_10_ would form nano-aggregates by hydrophobic interaction between grafted CAs, encapsulating Dox molecules inside of hydrophobic nano-aggregates core. In addition, Dox molecules also partially would be loaded inside of branched PEI chains according to the previous report [27].

In order to examine the formation of MCPEI-CA_10_ nano-aggregates, their Z-average sizes and Zeta-potential values were measured by Zeta-sizer.

**Figure 2:**
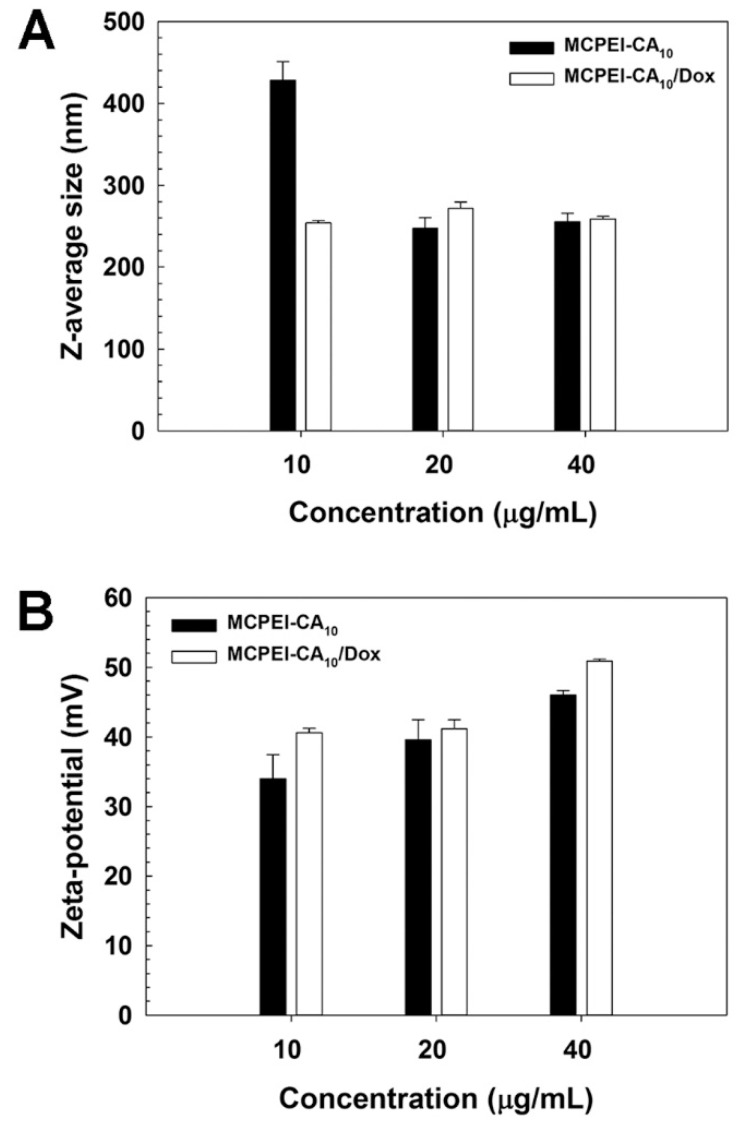
Z-average sizes (**A**) and Zeta-potential values (**B**) of cholic acid-conjugated methylcellulose-polyethylenimines (MCPEI-CA)_10_ nano-aggregates and MCPEI-CA_10_/Dox (Doxorubicin) nano-aggregates.

It was found that MCPEI-CA_10_ could form nano-aggregates with 428.2 nm diameter at 10 μg/mL, which was decreased to 253.9 nm after Dox encapsulation (Figure 2A). At higher concentrations, nano-aggregates with similar sizes (about 247–272 nm) were formed regardless of Dox encapsulation. This result means that primarily, MCPEI-CA_10_ could form nano-aggregates by hydrophobic interaction of CA moieties and that Dox molecules could facilitate the formation of compact nano-aggregates via their innate hydrophobicity and other interactions such as hydrogen bonds. In the case of Zeta-potential values, MCPEI-CA_10_ nano-aggregates showed 34.0 mV at 10 μg/mL, which mounted with the increase of concentration, up to 46.0 mV at 40 μg/mL (Figure 2B). It is assumed that positive Zeta-potential values of MCPEI-CA_10_ nano-aggregates would be due to the cationic amine moieties of PEI exposed on the surface of the nano-aggregates. After Dox encapsulation, their Zeta-potential values were increased to 40.6 mV at 10 μg/mL and also showed the proportional behaviors with the concentration. It was thought that positive charges of Dox by protonation of its amines would contribute to the increase of Zeta-potential values.

The morphology of MCPEI-CA_10_ nano-aggregates was examined by EF-TEM.

**Figure 3:**
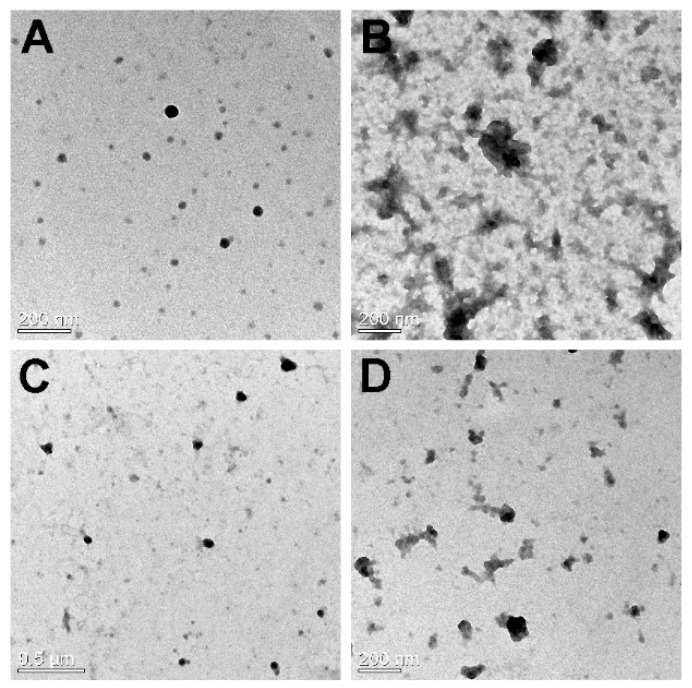
TEM images of cholic acid-conjugated methylcellulose-polyethylenimines (MCPEI-CA)_10_ nano-aggregates (**A**) 10 μg/mL, (**C**) 20 μg/mL, and MCPEI-CA_10_/Dox (Doxorubicin) nano-aggregates (**B**) 10 μg/mL, (**D**) 20 μg/mL.

In Figure 3, it was observed that MPEI-CA_10_ could form spherical nano-aggregates with about 50 nm diameter at both 10 and 20 μg/mL. However, MCPEI-CA_10_/Dox nano-aggregates showed relatively irregular and bumpy structures with similar sizes to MCPEI-CA_10_ nano-aggregates. It was thought that releasing Dox from the nano-aggregates during the sample preparation step would induce the irregular structures and background pattern. Smaller nano-aggregates sizes observed by TEM than hydrodynamic sizes measured by Zeta-sizer (dynamic light scattering) is probably due to the dry TEM condition, in comparison with the aqueous condition.

Dox release behavior of MCPEI-CA_10_ nano-aggregates was investigated at 37 °C (Figure S3). In both physiological condition (pH 7.4) and acidic endosomal condition (pH 5.4), Dox was released rapidly until 2 h, showing the initial burst release. During incubation time from 2 h to 24 h, Dox release was gradually increased up to about 24.8% at pH 5.4 and 15.8% at pH 7.4. Facilitated Dox release at pH 5.4 was thought to be due to the increase of Dox water solubility and electrostatic repulsion with cationic MCPEI-CA_10_ by protonation of Dox amine moieties.

### 3.3. Molecular Conformation of Polymers

**Figure 4:**
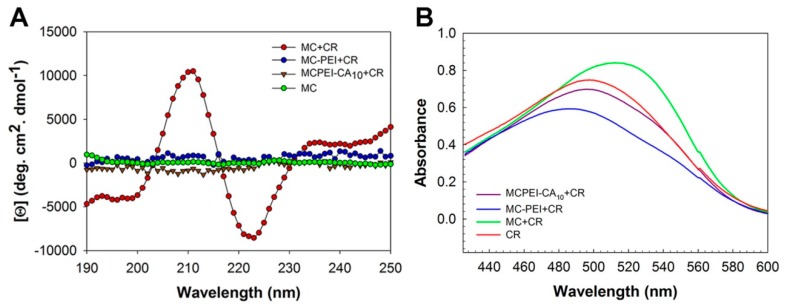
Circular dichroism (CD) spectra of Congo red (CR) in the polymer aqueous solutions (**A**) and CR absorbance measurement results in the polymer aqueous solutions (**B**).

Effect of CA conjugation to conformation of MC was investigated by circular dichroism (CD) and CR absorbance measurement. It has been already reported that MC would have at least partially helical conformation in dilute aqueous solution by induced CD (ICD) analysis [25]. Complexation between chiral and achiral molecules could give rise to ICD of the achiral counterparts, indicating the absolute configuration of the chiral molecules and their orientation relative to each other in the complex [28]. Congo red (CR), a dye molecule with a strong affinity to cellulose molecules, was employed to introduce chromophores for ICD because MC lacks accessible absorption bands. As shown in Figure 4A, the CD spectrum of MC is featureless because of the absence of absorption in this wavelength region, as expected. The CD spectrum of CR is also known to show no specific peaks due to its symmetric molecular structure [25]. However, after binding of CR to MC, induced CD spectra appeared, indicating the induced asymmetric orientation upon binding to MC chains with helical conformation. Interestingly, both MC-PEI and MCPEI-CA_10_ showed featureless CD spectra even in the presence of CR. It may be due to the reduced binding of CR molecules to MC-PEI and MCPEI-CA backbone by graft of bulky PEI moieties or loss of their helical conformation by conjugation of hydrophilic PEI moieties.

In order to examine the binding behavior of CR to the polymers, absorbance of CR in the polymer solution was measured. In general, it is known that maximum absorption of dye molecules can be shifted due to the formation of characteristic aggregation by their binding to polymer backbones [29]. After binding to polymer backbones, head-to-tail stacking of dye molecules (J-aggregates) can lead to a red-shift, while their parallel stacking can lead to a blue-shift (H-aggregates) [30,31]. In Figure 4B, absorbance peak of CR (λ_max_: 500 nm) was red-shifted (512 nm) in MC condition, suggesting the binding of CR along with MC chain (J-aggregates). In the case of MC-PEI, absorbance peak of CR was blue-shifted (486 nm). Bulky PEI moieties of MC-PEI backbone would cause the change of CR binding behavior to MC backbone (H-aggregates), finally inducing the shift of CR absorption. CR absorbance peak in MCPEI-CA_10_ solution was also weakly blue-shifted (496 nm), probably due to the reduced binding of CR to MCPEI-CA_10_ by nano-aggregates formation.

From these ICD and absorbance measurement results, it was found that CR molecules could bind to MC-PEI and MCPEI-CA_10_ backbones and that helical conformation of MC would be converted to optically inactive random coil conformation by PEI and CA conjugation.

### 3.4. In vitro Cell Experiments

Cytotoxicity of MCPEI-CAs was assessed by MTT assay in HeLa and A549 cells.

**Figure 5:**
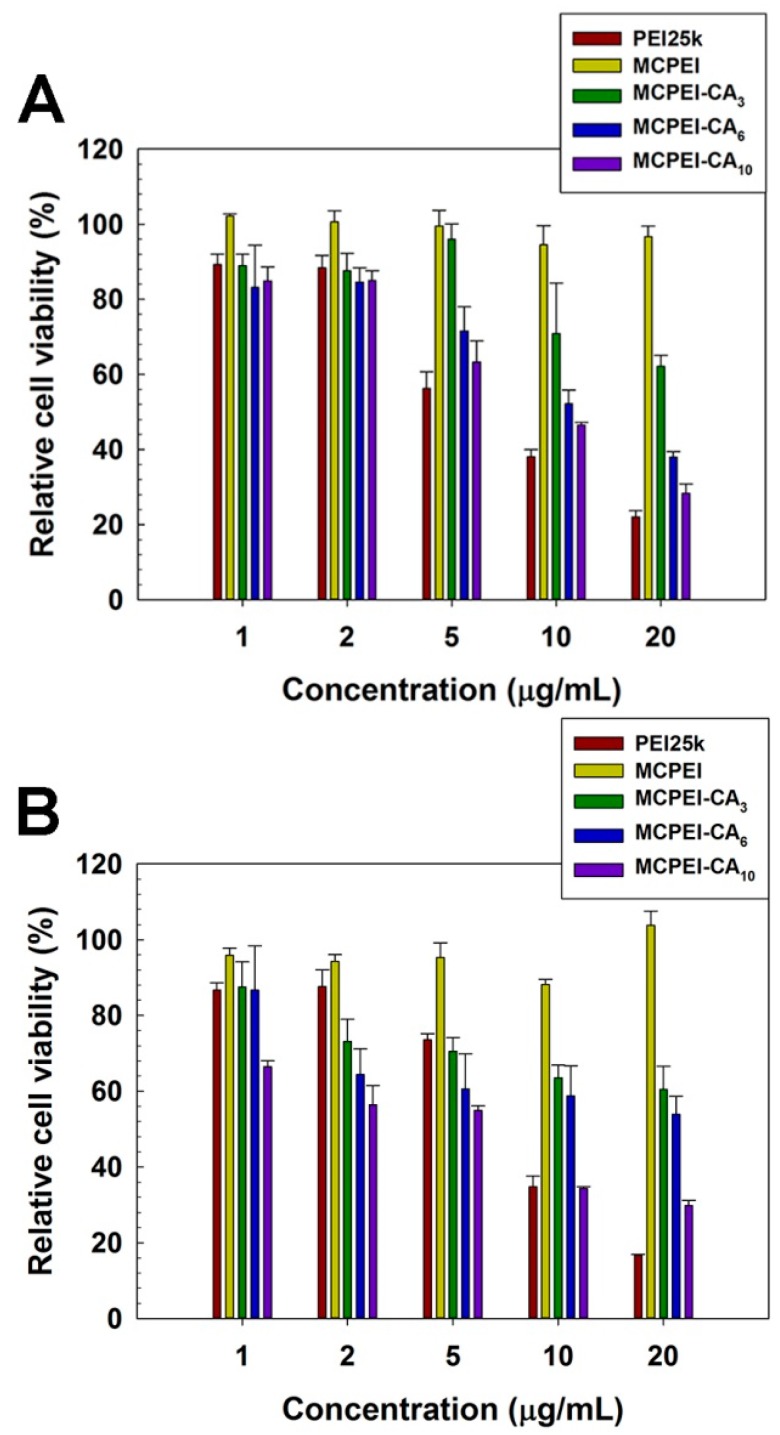
MTT assay results in HeLa cells (**A**) and A549 cells (**B**).

In Figure 5, PEI25k-treated cells showed a severe decrease of cell viability according to the increase of concentration, which means high cytotoxicity of PEI25k. Contrary to PEI25k, MC-PEI-treated cells showed high cell viability (>95% at 20 μg/mL), indicating its minimal cytotoxicity. In the case of MCPEI-CAs, their cytotoxicity increased with the increase of concentration and CA graft degree in both cell lines. MCPEI-CA_10_-treated cells showed about 30% cell viability at 20 μg/mL. Considering minimal cytotoxicity of their constituents, MC-PEI and CA, it was suggested that increased cytotoxicity of MCPEI-CAs would be derived from the specific molecular structures of MCPEI-CAs.

**Figure 6:**
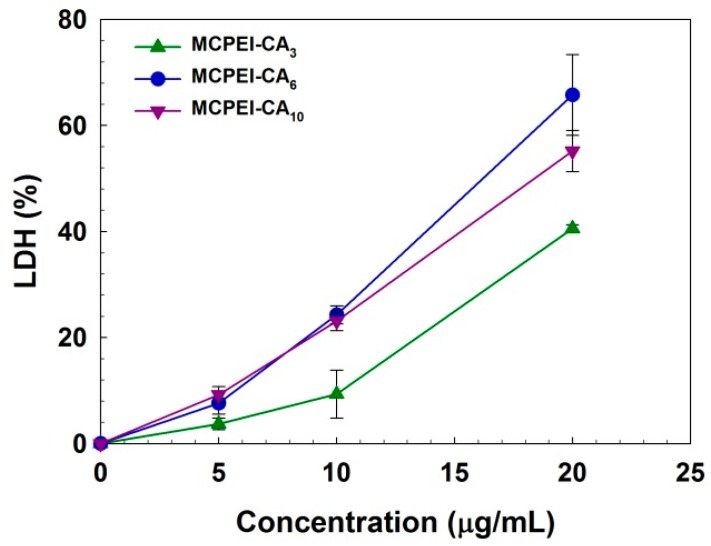
Lactate dehydrogenase (LDH) assay result in A549 cells.

In order to examine the interaction of MCPEI-CAs with cellular membrane, LDH assay was performed in A549 cells. LDH, a cytoplasmic enzyme catalyzing the reversible conversion of lactate to pyruvate, can be released from the cytoplasm by cellular membrane damage [32]. As shown in Figure 6, released LDH level was increased with the increase of concentration and CA graft degree of MCPEI-CAs. At 20 μg/mL, MCPEI-CA_3_, MCPEI-CA_6_, and MCPEI-CA_10_ showed 40.6%, 65.8%, and 55.2% LDH level, respectively. Therefore, it was thought that strong interaction of MCPEI-CA nano-aggregates with cellular membrane, via change of molecular structure by hydrophobic CA graft, would induce the damage to cellular membrane, finally leading to the cytotoxicity.

**Figure 7:**
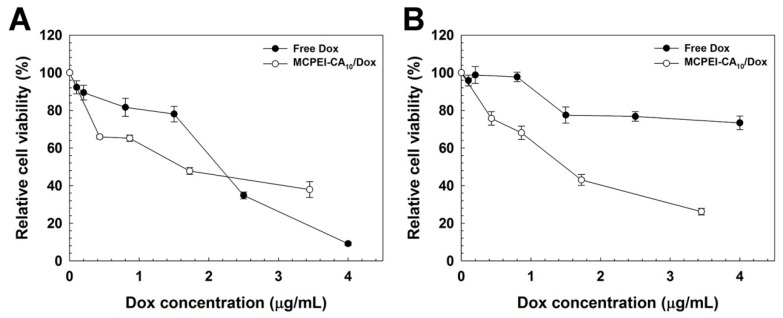
Anticancer activity result by MTT assay in HeLa cells (**A**) and A549 cells (**B**).

Then, the capability of MCPEI-CA nano-aggregates for drug delivery systems was investigated by measurement of anticancer activity of MCEI-CA/Dox nano-aggregates (Figure 7).

In HeLa cells (Figure 7A), MCPEI-CA_10_-treated cells showed higher cancer cell-killing activity than free Dox at low concentrations up to about 2 μg/mL, probably due to the combined effect of anticancer activity of encapsulated Dox and cytotoxicity of MCPEI-CA_10_. The cell viability decreased with the increase of concentration (at 3.4 μg/mL Dox concentration, 37.9% cell viability). Although MCPEI-CA_10_/Dox nano-aggregates displayed considerable anticancer activity, it was lower than that of free Dox at high concentration. However, in A549 cells (Figure 7B), cell viability of free Dox-treated cells was maintained as 73.3% even at a high concentration of 4 μg/mL, contrary to HeLa cell result. It would be due to the multidrug-resistance (MDR) activity of A549 cells [33,34], which could export the internalized Dox molecules by transporter proteins such as P-glycoprotein [35]. However, MCPEI-CA_10_-treated cells showed concentration-dependent decrease of cell viability and much higher decrease of cell viability than free Dox-treated cells, which suggests that MCPEI-CA_10_ nano-aggregates would deliver Dox molecules into cells, overcoming the MDR effect.

## 4. Conclusions

Cholic acid-conjugated methylcellulose-polyethylenimines (MCPEI-CAs) were synthesized and characterized for drug delivery systems. Molecular conformation change of MC derivatives by chemical modification (MC: helical structure, MC-PEI: random coil structure) was revealed. MCPEI-CA_10_ could form positively charged and spherical nano-aggregates by hydrophobic interaction of CA moieties, which was suitable for cellular uptake. MCPEI-CA_10_ could encapsulate the anticancer drug, doxorubicin (Dox), and its release was facilitated in acidic condition. Cytotoxicity of MCPEI-CAs increased with the increase of CA graft degrees, probably due to the cellular membrane disruption. MCPEI-CA_10_/Dox nano-aggregates showed concentration-dependent anticancer activity, which could overcome the multidrug resistance of cancer cells. From these results, it was concluded that MCPEI-CA_10_ nano-aggregates possessed a potential for drug delivery systems.

## Figures and Tables

**Table 1:** The chemical properties of cholic acid-conjugated methylcellulose-polyethylenimines (MCPEI-CAs).

	[PEI]:[CA] (Feed Ratio)	[PEI]:[CA] (^1^H NMR)
MCPEI-CA_3_	1:3.6	1:1.74
MCPEI-CA_6_	1:6.0	1:2.98
MCPEI-CA_10_	1:10.0	1:3.65

## Supplementary Materials

The following are available online at https://www.mdpi.com/2079-4991/9/3/459/s1, Figure S1: Synthesis scheme of MCPEI-CA, Figure S2: FT-IR spectra of MC-PEI and MCPEI-CAs, Figure S3: Dox release profile of MCPEI-CA_10_ nano-aggregates at pH 5.4 and 7.4.
