# Supplementary material for: Cholic Acid-Conjugated Methylcellulose-Polyethylenimine Nano-Aggregates for Drug Delivery Systems
Source: Nanomaterials (Basel). 2019 Mar 19;9(3):459. doi: 10.3390/nano9030459 (PMC6474074; doi:10.3390/nano9030459)
Supplement: Supplementary file 1 [file nanomaterials-09-00459-s001.pdf]

*Supplementary materials*

# Cholic Acid-conjugated Methylcellulose-Polyethylenimine Nano-aggregates for Drug Delivery Systems

**Taewan Kim [1,†], Jaehong Park [1,†] and Tae-il Kim [1,2,*]**

[1] Department of Biosystems & Biomaterials Science and Engineering, College of Agriculture and Life Sciences, Seoul National University, 1 Gwanak-ro, Gwanak-gu, Seoul, 08826, Korea; tewhan32321@naver.com (T.K.); pjh0520@snu.ac.kr (J.P.)

[2] Research Institute of Agriculture and Life Sciences, Seoul National University, 1 Gwanak-ro, Gwanak-gu, Seoul, 08826, Korea

† These authors contributed equally to this work

* Correspondence: seal1004@snu.ac.kr

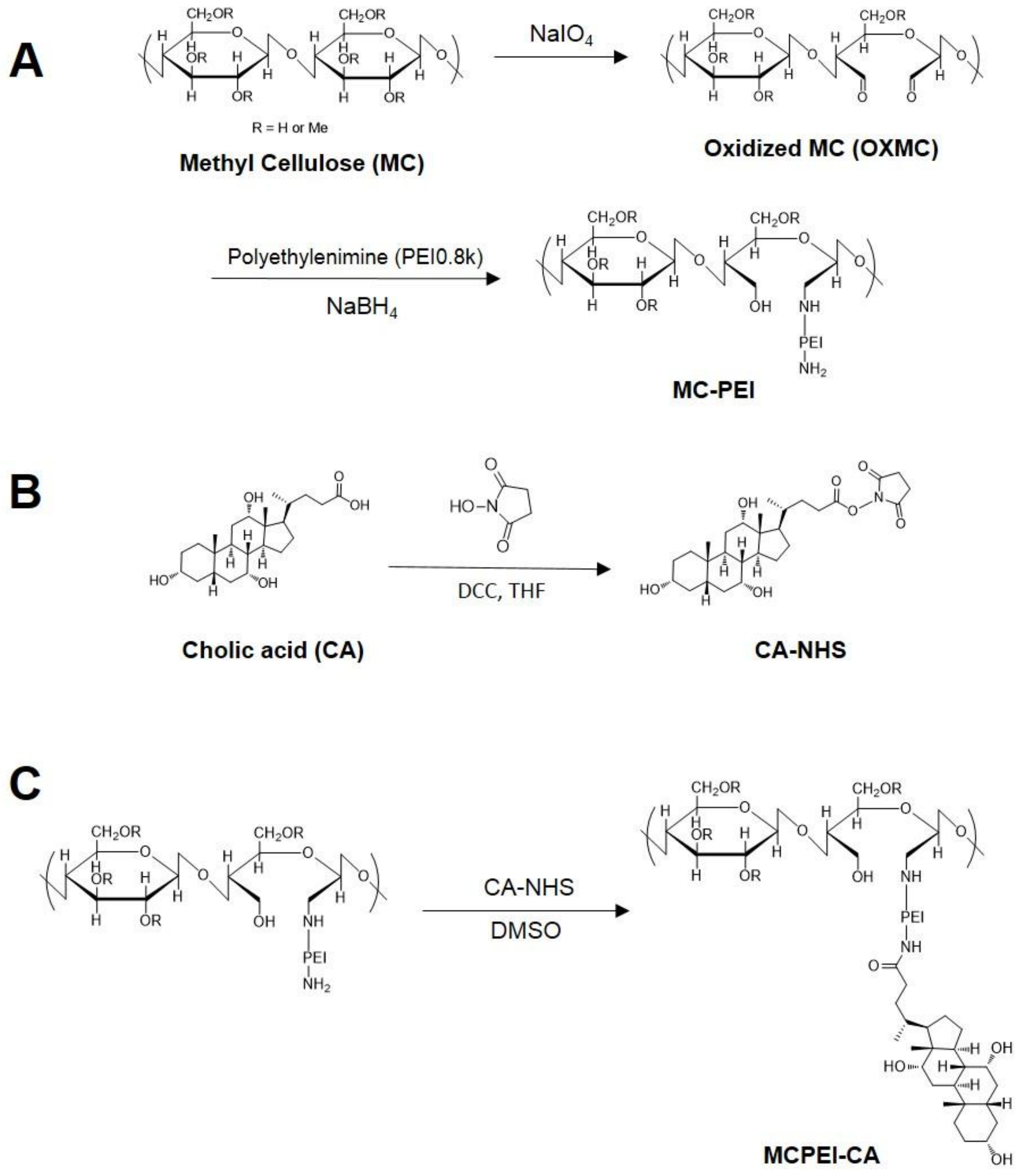


**Figure 1.** Synthesis scheme of MCPEI-CAs.

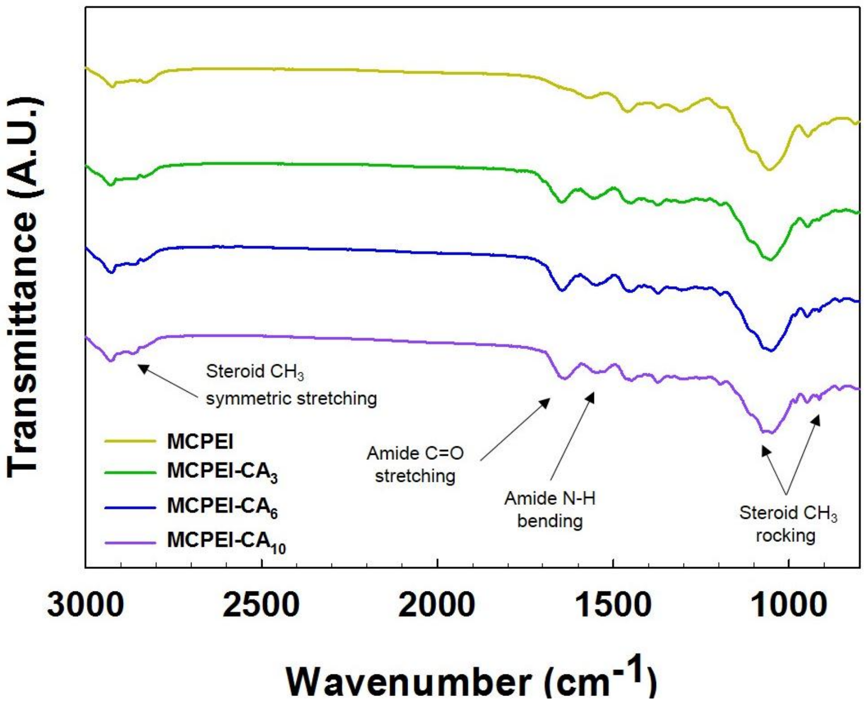


**Figure 2.** FT-IR spectra of MC-PEI and MCPEI-CAs.

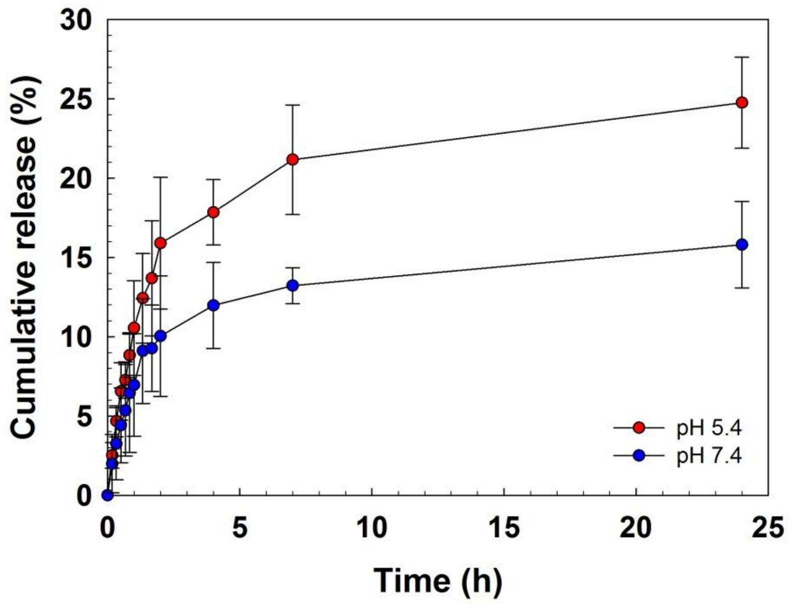


**Figure 3.** Dox release profile of MCPEI-$CA_{10}$ nano-aggregates at pH 5.4 and 7.4.
